# Plasma Mucin-1 as a Potential Biomarker for Diabetic Peripheral Neuropathy in Type 2 Diabetes

**DOI:** 10.3390/biom16010128

**Published:** 2026-01-12

**Authors:** Jae-Hyung Park, Thi Nhi Nguyen, Hye Min Shim, Gyeong Im Yu, Junho Kang, Eun Yeong Ha, Hochan Cho

**Affiliations:** 1 Department of Physiology, School of Medicine, Keimyung University, Daegu 42601, Republic of Korea; physiopark@kmu.ac.kr (J.-H.P.); nguyenthinhiydh@gmail.com (T.N.N.); hmshim1110@gmail.com (H.M.S.); rki0411@hanmail.net (G.I.Y.); 2 Department of Research, Keimyung University Dongsan Medical Center, Daegu 42601, Republic of Korea; junho6399@dsmc.or.kr; 3 Department of Internal Medicine, Keimyung University School of Medicine, Daegu 42601, Republic of Korea; 240012@dsmc.or.kr

**Keywords:** diabetes mellitus, type 2, diabetic neuropathies, peripheral nerve injuries, mucin-1, biomarkers, CA15-3

## Abstract

Background: Diabetic peripheral neuropathy (DPN) is a major complication of type 2 diabetes mellitus (T2D) that reduces quality of life and increases the risk of foot ulcers and amputations. Early detection is essential, and blood-based biomarkers may support improved screening and timely intervention. This study aimed to identify novel circulating biomarkers for the identification of DPN in patients with T2D. Methods: In the screening phase, plasma samples from 43 participants (10 healthy volunteers [HV], 20 T2D without complications, and 13 T2D with DPN) were analyzed using an antibody array targeting 310 proteins. Thirteen differentially expressed proteins were identified, and six hub proteins were selected through bioinformatic analysis. In the validation phase, plasma concentrations of the six proteins were measured by ELISA in 252 subjects (100 HV, 97 T2D without complications, and 55 T2D with DPN). Mucin-1 expression in sciatic nerves was further evaluated in db/db mice. Results: Of the six hub proteins (TGFB1, MUC1, PF4, IL2RA, SELL, B2M), only mucin-1 showed a significant increase in the DPN group. Plasma mucin-1 positively correlated with MNSI scores and negatively with motor and sensory nerve conduction velocities. In db/db mice, sciatic nerve mucin-1 expression was elevated, while CD31 expression was reduced. Conclusions: Plasma mucin-1 is strongly associated with DPN in both humans and animals and may serve as a promising biomarker for the screening and early identification of DPN.

## 1. Introduction

Diabetic peripheral neuropathy (DPN) is a common complication of diabetes with a multifactorial pathophysiology involving metabolic dysregulation, neuroinflammation, oxidative stress, and microvascular impairment, ultimately leading to progressive peripheral nerve injury [1,2]. In well-standardized epidemiological studies incorporating both clinical and electrophysiological evaluations, diabetic peripheral neuropathy is estimated to affect approximately 20–50% of adult patients with diabetes [3,4]. DPN typically develops gradually, beginning with mild symptoms such as tingling or numbness in the extremities and potentially progressing to severe pain, muscle weakness, and sensory loss [5]. Advanced stages can lead to foot ulcers, infections, and even amputations, while autonomic neuropathy may result in cardiovascular or gastrointestinal dysfunction [6]. Although glycemic control and symptomatic treatments such as pain relief medications and physical therapy are used to manage DPN, effective strategies to reverse established nerve damage are still lacking [1]. Therefore, timely identification of DPN at clinically detectable stages is critical for preventing progression and serious outcomes [3].

The diagnosis of DPN relies on a combination of patient history, neurological examination, and functional tests, including monofilament testing, electromyography, and nerve conduction studies [7]. However, these diagnostic methods have several limitations: nerve conduction studies and electromyography can be uncomfortable and are not suitable for routine screening, while questionnaires such as the Michigan Neuropathy Screening Inventory (MNSI) may be less reliable in elderly patients or those with cognitive impairment [8]. Moreover, early neuropathy can remain asymptomatic, delaying detection [9].

To overcome these challenges, the development of blood-based biomarkers has been proposed as an important complementary strategy [10,11]. Although various biomarkers—such as inflammatory mediators, oxidative stress markers, and neurotrophic factors—have been investigated, their diagnostic reliability and reproducibility remain limited [12]. Mucin-1 (MUC1), also known as CA15-3, is a high-molecular-weight transmembrane glycoprotein expressed on epithelial surfaces and has been identified as a structural component of the perineurium in peripheral nerves [13]. The perineurium plays a critical role in maintaining nerve barrier integrity and is known to undergo structural remodeling and proliferation in diabetic peripheral neuropathy [14]. Beyond its barrier-related function, mucin-1 has been implicated in inflammatory signaling, oxidative stress responses, and endothelial dysfunction, processes that are closely linked to microvascular and neurovascular injury in diabetes [15]. Therefore, alterations in mucin-1 expression may reflect perineurial structural remodeling and metabolic stress in diabetic nerves and may be mirrored by increased circulating levels, supporting its biological plausibility as a blood-based biomarker for DPN.

In this study, we aimed to identify circulating biomarkers for the identification and screening of DPN in patients with type 2 diabetes (T2D). Protein profiles were compared among healthy volunteers (HV), T2D patients without complications, and T2D patients with DPN using antibody array analysis. Candidate biomarkers were further validated by enzyme-linked immunosorbent assay (ELISA). Finally, to support translational relevance, we evaluated the expression of mucin-1 within the sciatic nerves of 30-week-old db/db mice, a well-established model of diabetic neuropathy [16].

## 2. Materials and Methods

### 2.1. Human Subjects and Research Design

Participants for both discovery and validation phases were prospectively enrolled at Keimyung University Dongsan Hospital (Daegu, Republic of Korea) between July 2019 and August 2023. The study protocol (IRB No. 2018-05-058) was approved by the institutional review board, and written informed consent was obtained from all participants prior to inclusion. Subjects were categorized into three groups according to the American Diabetes Association (ADA) criteria [17]: (1) healthy volunteers (HV), (2) patients with type 2 diabetes (T2D) without microvascular complications, and (3) T2D patients with diabetic peripheral neuropathy (DPN) as the sole complication. Patients in the DPN group were required to have clinically and electrophysiologically confirmed diabetic peripheral neuropathy, whereas patients in the T2D without DPN group had no clinical or electrophysiological evidence of neuropathy or other diabetic microvascular complications. For the antibody array screening, 10 HV, 20 T2D without complications, and 13 T2D with DPN were examined. The validation cohort comprised 100 HV, 97 T2D without DPN, and 55 T2D with DPN. Exclusion criteria included the presence of other potential causes of peripheral neuropathy (such as alcohol abuse, vitamin B12 deficiency, autoimmune neuropathies, or neurotoxic medication use), a known history of malignancy, chronic liver disease, severe renal impairment, acute infection, or active systemic inflammatory or fibrotic disorders that could influence circulating biomarker levels. Patients with diabetic microvascular complications other than DPN, including diabetic nephropathy and retinopathy, were also excluded to ensure that DPN was the sole complication evaluated. Peripheral venous blood samples were collected in the morning after an overnight fast of 8–10 h using EDTA-containing tubes. Plasma was separated by centrifugation at 1500× *g* for 10 min at 4 °C within 2 h of collection, aliquoted to avoid repeated freeze–thaw cycles, and stored at −80 °C until analysis. DPN was diagnosed using a combination of clinical assessment and electrophysiological evaluation. Clinical symptoms were assessed using the Michigan Neuropathy Screening Instrument questionnaire (MNSI-Q), with a score ≥ 3 considered indicative of peripheral neuropathy. Electrophysiological studies of the lower extremities included measurements of motor nerve conduction velocity (MNCV), sensory nerve conduction velocity (SNCV), and sensory nerve action potential (SNAP). DPN was defined by the presence of abnormal nerve conduction parameters, including reduced conduction velocity and/or decreased amplitude, in at least two nerves, one of which was required to be a sensory nerve.

### 2.2. Antibody Array Screening and ELISA Validation

Plasma proteins were extracted using a proprietary buffer (Full Moon BioSystems, Sunnyvale, CA, USA). Relative levels of 310 plasma proteins were quantified with the Cytokine Profiling Antibody Array kit (Full Moon BioSystems, SCK100). The assay workflow and data normalization procedures were conducted as described previously with minor modifications [18]. Candidate proteins were quantified using ELISA or multiplex bead-based assays. IL-2RA, L-selectin, platelet factor-4, and TGF-β1 were measured using magnetic bead multiplex assays (R&D Systems, Minneapolis, MN, USA). β-2-microglobulin (RayBiotech, Peachtree Corners, GA, USA) and mucin-1 (CIS Bio International, Saclay, France) were determined using specific ELISA kits.

### 2.3. Bioinformatic Analyses

Differentially expressed proteins showing ≥2-fold changes were functionally annotated using the DAVID 6.8 platform for Gene Ontology (GO) and KEGG pathway enrichment. Protein–protein interaction (PPI) networks were constructed through the STRING database integrated in Cytoscape 3.10.3 (confidence score ≥ 0.4).

### 2.4. Animal Experiments

Male db/db mice and age-matched heterozygous littermates (4 weeks old; Jung-Ang Experimental Animals, Seoul, Republic of Korea) were used in this study. Mice were housed under specific pathogen-free conditions in a temperature-controlled environment (22 ± 2 °C) with a 12 h light/dark cycle and had ad libitum access to standard chow diet (Research Diet, New Brunswick, NJ, USA) and water. For each experimental time point (4, 10, 18, 24, and 30 weeks of age), seven mice per group (db/db and control) were used for behavioral and molecular analyses. An additional cohort of seven mice per group at 30 weeks of age was used for immunofluorescence-based sciatic nerve imaging. Animals were allocated to experimental groups based on genotype. Thermal nociception was assessed using the Hargreaves test. After a 20 min acclimatization period, infrared light was focused on the plantar surface of the hind paw, and paw withdrawal latency was automatically recorded with a cut-off time of 15 s to prevent tissue damage. Measurements were repeated three times with at least 5 min intervals over three consecutive days. Mechanical sensitivity was evaluated using the von Frey filament test under similar conditions. All behavioral assessments and histological analyses were performed by investigators blinded to group allocation. After an overnight fast of approximately 8 h, blood samples and sciatic nerve tissues were collected. An 8 h fasting period was selected to minimize metabolic stress and potential adverse effects in aged db/db mice. The animal study protocol was approved by the Institutional Review Board of Keimyung University Animal Ethics Committee (protocol code KM-2021-19, approved on 3 September 2021).

### 2.5. Quantitative Real-Time PCR

Total RNA was extracted from sciatic nerves using TRIzol (Sigma-Aldrich, St. Louis, MO, USA). cDNA synthesis was performed using the High-Capacity cDNA Reverse Transcription Kit (Applied Biosystems, Foster City, CA, USA). Quantitative PCR was conducted using SYBR Green reagent on a 7500 Real-Time PCR System (Applied Biosystems). Gene expression was calculated by the 2^−ΔΔCt^ method with GAPDH as a reference.

### 2.6. Immunofluorescence Staining

Fluorescence images were captured using a confocal laser scanning microscope (Stellaris 5, Leica Microsystems, Wetzlar, Germany). For this experiment, formalin-fixed, paraffin-embedded sciatic nerve sections (4 µm) were first incubated with primary antibodies against CD31 (Santa Cruz Biotechnology, Santa Cruz, CA, USA) and mucin-1 (Abcam, Cambridge, MA, USA). After washing, Alexa Fluor 488- or 546-labeled secondary antibodies (Thermo Fisher Scientific, Waltham, MA, USA) were applied.

### 2.7. Statistical Analysis

Between-group comparisons used Student’s *t*-test or one-way ANOVA followed by Tukey’s post hoc test. Continuous variables were expressed as mean ± standard error. Correlations were assessed by Pearson’s analysis. Logistic regression was applied to identify independent predictors of DPN, adjusting for diabetes duration and medication history (lipid-lowering agents, NSAIDs, and antidiabetic drugs). The diagnostic ability of the model was assessed by plotting receiver operating characteristic (ROC) curves and calculating the area under the curve (AUC). Statistical analyses were conducted with SPSS 27.0 (IBM, Armonk, NY, USA). *p* < 0.05 was considered significant.

## 3. Results

### 3.1. Identification of Plasma Proteins for DPN in Patients with T2D

A total of 43 participants were included in the screening experiment: 10 HV, 20 T2D patients without DPN, and 13 T2D patients with DPN. Baseline characteristics are summarized in Table 1. Significant differences were observed in fasting glucose, HbA1c, fasting insulin, Homeostatic Model Assessment for Insulin Resistance (HOMA-IR), urine microalbumin–creatinine (M/C) ratio, and duration of diabetes among the three groups, whereas no differences were noted in age, body weight, BMI, waist circumference, estimated glomerular filtration rate (eGFR), creatinine, aspartate aminotransferase (AST), alanine transaminase (ALT), triglyceride, or cholesterol levels.

**Figure 1:**
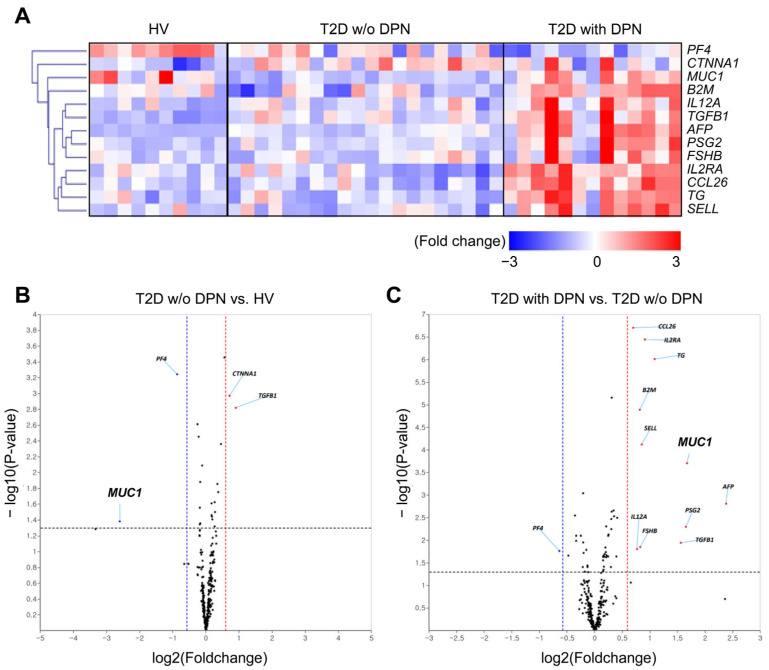
Distinct features of circulating proteins expressed by antibody array. (**A**) Heatmap of protein profile using blood antibody array between HV, T2D without DPN and T2D with DPN. The color scale ranges from blue to red, indicating low expression to high expression (log2 fold change from −3 to 3). Volcano plots of protein expression of T2D without DPN versus HV (**B**) and T2D with DPN versus T2D without DPN (**C**) were constructed using log2 fold-change values and *p*-values. Black dotted horizontal lines indicate the threshold for statistical significance (*p* < 0.05). The vertical lines correspond to 1.5-fold up-regulation (red) and down-regulation (blue), and the horizontal line represents a *p*-value. While red spots represent proteins with increased expression, blue counterparts indicate decreased expression proteins. HV, healthy volunteers; T2D, type 2 diabetes; w/o, without; DPN, diabetic peripheral neuropathy.

Plasma protein profiles were analyzed using an antibody array targeting 310 proteins (Supplementary Table S1). Thirteen proteins that differed by ≥2-fold in at least one comparison between groups were identified (Figure 1A). The distribution of significantly altered proteins is shown in volcano plots (Figure 1B,C). Proteins with an absolute log2 fold change ≥2 and *p* ≤ 0.05 were classified as significantly differentially expressed. Compared with HV, the T2D without DPN group showed upregulation of CTNNA1 and TGFB1 and downregulation of PF4 and MUC1. In contrast, comparison of T2D with DPN versus T2D without DPN revealed 13 differentially expressed proteins, including 12 upregulated (CCL26, IL2RA, TG, B2M, SELL, MUC1, AFP, IL12A, FSHB, PSG2, TGFB1, and one duplicate removed) and one downregulated (PF4).

### 3.2. Functional Enrichment Analysis of Antibody Array

Functional enrichment analysis was performed to explore the biological roles of the 13 proteins differentially expressed between T2D without DPN and T2D with DPN groups (Supplementary Figure S1A). Gene ontology analysis revealed enrichment of biological processes such as epithelial cell proliferation, cell killing, and the extrinsic apoptotic signaling pathway (Supplementary Figure S1B). KEGG pathway analysis indicated that cytokine–cytokine receptor interaction and the Hippo signaling pathway were primarily associated with DPN (Supplementary Figure S1C).

**Figure 2:**
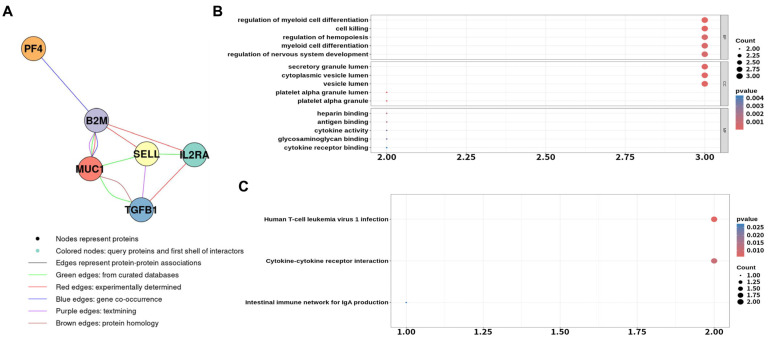
Functional enrichment analysis of selected proteins. (**A**) PPI network of the six most significant proteins derived from antibody array analysis. The nodes indicate the proteins that connect with others through edges, showing an interaction between linked proteins. Enrichment analysis with GO (**B**) and KEGG (**C**) pathway analysis of top six proteins. Top five GO terms of three criteria, BP, CC, and MF (**B**), and top three KEGG pathways (**C**) are illustrated, respectively. The count value on x-axis is the number of significant proteins over the total proteins involved in the GO or pathway. PPI, protein–protein interaction; GO, gene ontology; MF, molecular function; CC, cellular component; BP, biological process.

Through node analysis, six hub proteins (TGFB1, MUC1, SELL, IL2RA, PF4, and B2M) were identified (Figure 2A). Further enrichment analysis of these hub proteins additionally highlighted biological processes related to nervous system development (Figure 2B), as well as KEGG pathways including cytokine–cytokine receptor interaction and the intestinal immune network for IgA production (Figure 2C). Consistently, plasma concentrations of TGFB1, MUC1, SELL, IL2RA, and B2M were relatively elevated in the T2D with DPN group compared with the T2D without DPN group, whereas PF4 was slightly decreased (Supplementary Figure S2).

### 3.3. Validation of Mucin-1 as a Potential Biomarker for DPN

**Figure 3:**
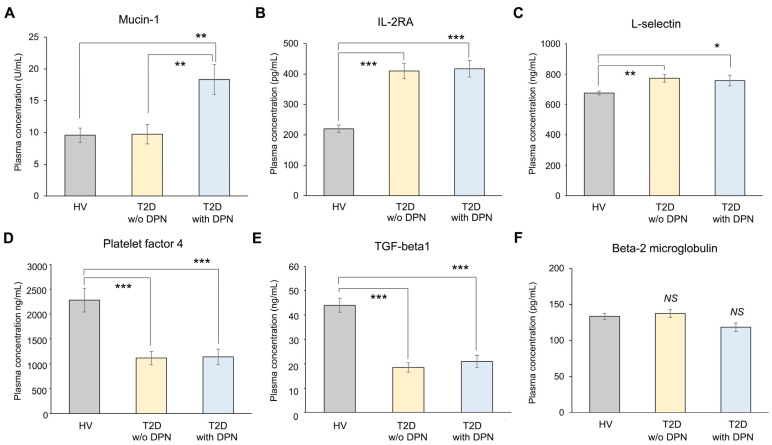
Concentrations of selected plasma proteins. Plasma Concentrations of mucin-1 (**A**), IL-2RA (**B**), L-selectin (**C**), platelet factor 4 (**D**), TGF-beta 1 (**E**), beta-2 microglobulin (**F**) were measured by ELISA kits in HV, T2D without DPN, and T2D with DPN. Data are presented as mean ± SEM (*n* = 100 for HV, *n* = 97 for T2D without DPN, and *n* = 95 for T2D with DPN). * *p* < 0.05, ** *p* < 0.01 and *** *p* < 0.001. HV, healthy volunteers; T2D, type 2 diabetes; w/o, without; DPN, diabetic peripheral neuropathy; NS, not significant.

To validate potential biomarkers, we recruited an independent cohort consisting of 100 HV, 97 T2D without DPN, and 55 T2D with DPN. Baseline characteristics are summarized in Table 2. Significant differences among the three groups were observed in fasting glucose, HbA1c, fasting insulin, HOMA-IR, M/C ratio, and duration of diabetes, whereas no significant differences were noted in age, body weight, eGFR, creatinine, AST, ALT, triglyceride, HDL, or LDL levels. Plasma concentrations of six candidate proteins were measured using ELISA (Figure 3). Among them, mucin-1 was the only protein that showed a significant difference between groups. A significant increase in plasma mucin-1 was observed among patients with DPN (17.76 ± 4.45 U/mL) compared with both the T2D without DPN group (9.66 ± 2.02 U/mL, *p* = 0.029) and the HV group (9.56 ± 2.13 U/mL, *p* = 0.021) (Figure 3A). In contrast, IL-2RA, L-selectin, PF4, and TGF-β showed significant changes in both diabetic groups compared with HV but did not differ between the two diabetic groups (Figure 3B–F).

### 3.4. Correlation Analysis of Mucin-1 with Clinical Parameters

In a comparison between the two diabetic groups (Table 2), there were no significant differences in clinical parameters except for the duration of diabetes. The MNSI score and plasma mucin-1 concentrations were significantly higher in the T2D with DPN group than in the T2D without DPN group, whereas MNCV, SNCV, and SNAP of the lower limb were significantly reduced (Table 3). Unadjusted Pearson correlation analysis demonstrated that plasma mucin-1 levels were positively correlated with MNSI score and duration of diabetes and negatively correlated with MNCV, SNCV, and SNAP of the lower limb (Table 4). No significant correlations were observed between mucin-1 and other metabolic indices, including BMI, fasting glucose, HbA1c, fasting insulin, HOMA-IR, or the M/C ratio.

**Figure 4:**
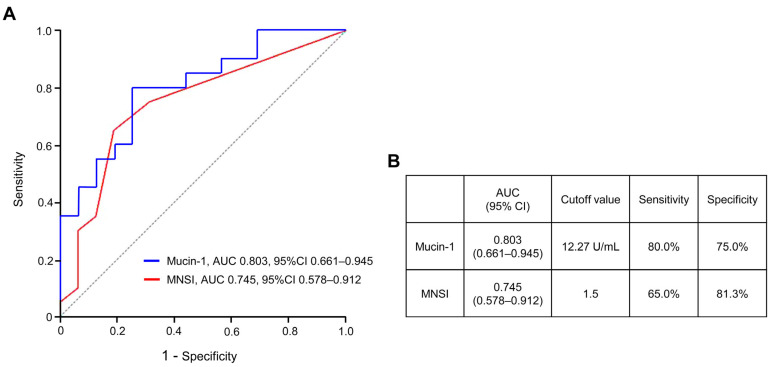
Receiver operating characteristic (ROC) curves of plasma mucin-1 and MNSI. (**A**) The ROC curves of plasma mucin-1 levels or MNSI scores for DPN compared with T2D without DPN. (**B**) The AUC, sensitivity, specificity and cutoff value of plasma mucin-1 levels or MNSI scores for DPN compared with T2D without DPN. The optimal cutoff point was selected at the highest sensitivity while maintaining high specificity. The black dotted diagonal line represents the reference line for random classification (AUC = 0.5). CI, confidence interval; AUC, area under the curve; MNSI, Michigan Neuropathy Screening Inventory; DPN, diabetic peripheral neuropathy.

To further evaluate whether mucin-1 was independently associated with DPN beyond the effect of diabetes duration, univariate and multivariate logistic regression models were constructed. After adjustment for diabetes duration, mucin-1, MNSI score, MNCV, SNCV, and SNAP remained independent predictors of clinically manifest DPN (Table 5). In addition, ROC curve analysis was performed to compare the diagnostic performance of mucin-1 and MNSI (Figure 4). Mucin-1 demonstrated a higher discriminative ability with an AUC of 0.803 (95% CI, 0.661–0.945) compared with the MNSI score (AUC = 0.745, 95% CI, 0.578–0.912). The optimal cut-off value of mucin-1 for predicting DPN was 12.27 U/mL, yielding a sensitivity of 80.0% and specificity of 75.0%, whereas the MNSI showed a sensitivity of 65.0% and specificity of 81.3%.

### 3.5. The Assessment of Mucin-1 Expression in Sciatic Nerve of T2D Mice

**Figure 5:**
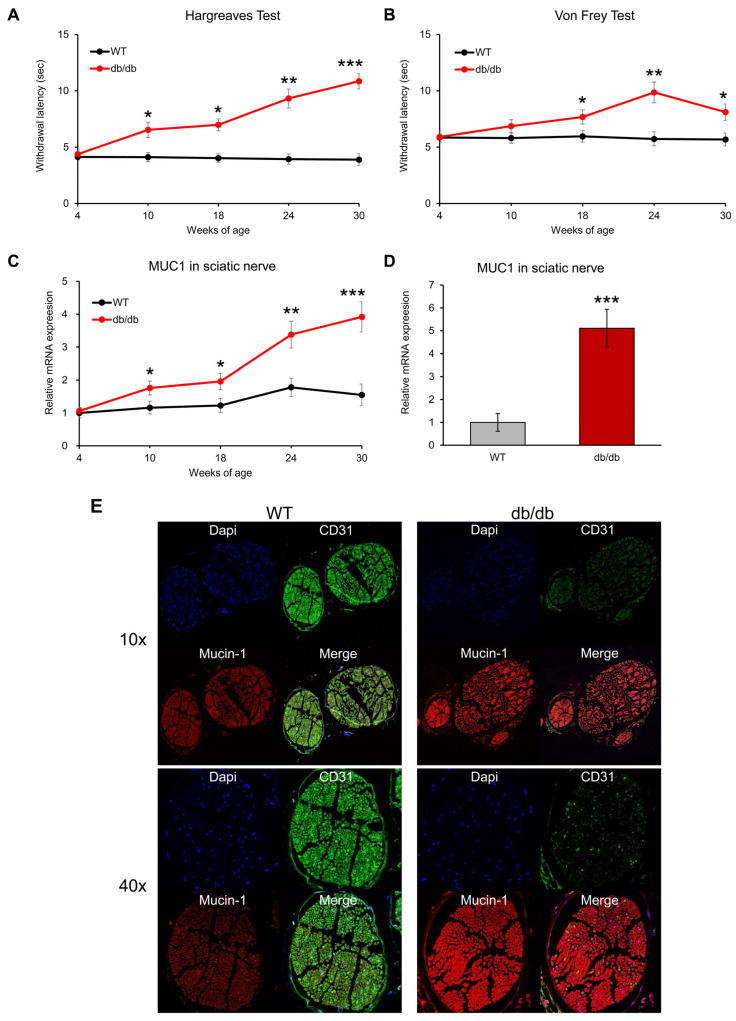
Time-dependent changes in sensory function and mucin-1 expression in the sciatic nerves of db/db mice. (**A**) Thermal withdrawal latency assessed by the Hargreaves test and (**B**) mechanical withdrawal threshold measured by the von Frey test in db/db mice and age-matched wild-type (WT) controls at 4, 10, 18, 24, and 30 weeks of age. (**C**) Relative MUC1 mRNA expression levels in sciatic nerves across age groups and (**D**) comparison of MUC1 mRNA expression between WT and db/db mice at 30 weeks of age. (**E**) Representative immunofluorescence images showing CD31 (green) and mucin-1 (red) expression in sciatic nerves from 30-week-old WT and db/db mice. Data are presented as mean ± SEM (*n* = 7 per group at each time point). * *p* < 0.05, ** *p* < 0.01, *** *p* < 0.001.

To assess the temporal progression of neuropathic changes, behavioral and molecular analyses were performed in db/db mice and age-matched controls at 4, 10, 18, 24, and 30 weeks of age (*n* = 7 per group at each time point). In the Hargreaves test, thermal withdrawal latency did not differ between groups at 4 weeks of age; however, db/db mice exhibited a significant increase beginning at 10 weeks (*p* = 0.042), with progressive prolongation observed at 18 (*p* = 0.024), 24 (*p* = 0.0089), and 30 weeks of age (*p* = 0.00092) (Figure 5A). In contrast, mechanical withdrawal thresholds assessed by the von Frey test remained comparable between groups until 10 weeks but became significantly elevated from 18 weeks onward (18 weeks, *p* = 0.037; 24 weeks, *p* = 0.0093; 30 weeks, *p* = 0.045), indicating a delayed but progressive impairment of mechanical sensitivity (Figure 5B). Consistent with these behavioral alterations, MUC1 mRNA expression in the sciatic nerves of db/db mice was significantly upregulated starting at 10 weeks of age (*p* = 0.046) and increased further with advancing age (18 weeks, *p* = 0.048; 24 weeks, *p* = 0.0076; 30 weeks, *p* = 0.00094), reaching approximately fivefold higher levels than controls at 30 weeks (Figure 5C,D). Immunofluorescence analysis of sciatic nerves from 30-week-old mice demonstrated reduced expression of the vascular marker CD31 [19] and marked accumulation of mucin-1 in db/db mice compared with wild-type controls (Figure 5E). Together, these findings indicate a time-dependent emergence and progression of sensory dysfunction accompanied by increasing perineurial mucin-1 expression in diabetic neuropathy.

## 4. Discussion

Early detection of DPN is crucial for mitigating disease progression and reducing its clinical burden, as timely intervention can significantly improve patient outcomes [20]. Several biomarkers reflecting underlying pathological mechanisms have been investigated, including inflammatory cytokines such as TNF-α and IL-6 [21], oxidative stress markers like advanced glycation end products (AGEs) [22] and malondialdehyde (MDA) [23], as well as metabolic alterations involving molecules such as nerve growth factor (NGF) [24] and homocysteine [25,26]. Additionally, neurofilament light chain (NFL) has emerged as a promising indicator of axonal injury, providing insights into the neurodegenerative processes that underlie DPN [27]. However, the insufficient specificity and sensitivity of these biomarkers in routine clinical settings underscore the critical need for further research to identify and validate novel blood biomarkers that could facilitate early and accurate diagnosis [11].

In this study, we comprehensively screened 310 plasma proteins in T2D patients with and without DPN and identified 13 candidates. Bioinformatic prioritization highlighted six hub proteins, among which mucin-1 was the only protein consistently elevated in patients with DPN compared with both HV and T2D without DPN. Validation using ELISA confirmed a significant increase in plasma mucin-1 in the DPN group. Importantly, plasma mucin-1 correlated positively with MNSI scores and diabetes duration and negatively with motor and sensory nerve conduction velocities, suggesting a strong association with clinical and electrophysiological measures of neuropathy. ROC analysis further demonstrated superior sensitivity of mucin-1 (80.0%) compared with MNSI (65.0%), supporting its potential utility as a screening biomarker. It should be noted that patients with DPN had a significantly longer duration of diabetes than those without DPN in both the screening and validation cohorts, which is an expected clinical characteristic given that the risk of neuropathy increases with cumulative glycemic exposure. Accordingly, the observed positive correlation between plasma mucin-1 levels and diabetes duration does not necessarily indicate a nonspecific effect of disease chronicity alone. Importantly, multivariate logistic regression analyses demonstrated that mucin-1 remained independently associated with DPN after adjustment for diabetes duration. Moreover, the significant associations between mucin-1 and objective neuropathy-related measures, including nerve conduction velocities and MNSI scores, support the interpretation that mucin-1 elevation reflects neuropathy-related structural and functional changes rather than prolonged diabetes duration per se. Nevertheless, longitudinal studies are warranted to further clarify the temporal relationship between diabetes duration, neuropathy progression, and circulating mucin-1 levels.

Mucin-1 is a high-molecular-weight transmembrane glycoprotein expressed on epithelial surfaces and, notably, in the perineurium of peripheral nerves, where it contributes to barrier integrity and regulation of the nerve microenvironment [13,15]. The perineurium plays a critical role in maintaining endoneurial homeostasis by protecting nerve fibers from mechanical stress and circulating metabolic or inflammatory insults. In diabetic peripheral neuropathy, perineurial thickening and structural remodeling are prominent pathological features [14,28,29], reflecting chronic exposure to hyperglycemia, oxidative stress, and microvascular dysfunction. Under these conditions, upregulation of mucin-1 may represent an adaptive response aimed at reinforcing perineurial barrier function; however, persistent metabolic and inflammatory stress may lead to excessive mucin-1 expression and subsequent leakage into the circulation. Recent high-resolution imaging studies have further demonstrated that diabetic neuropathy, particularly in distal sensory nerves, is characterized by microstructural alterations such as reduced axonal density and altered myelin content. These changes are detectable by diffusion-based MRI metrics, including fractional anisotropy, which correlate closely with histologically verified nerve fiber integrity, especially in the sural nerve that is selectively vulnerable in type 2 diabetes [30,31,32]. Although such imaging modalities were not applied in the present study, these findings provide an important structural context for interpreting the association between circulating mucin-1 levels and electrophysiological impairment, suggesting that mucin-1 elevation may reflect underlying microstructural nerve damage rather than purely functional abnormalities. Consistent with this hypothesis, our animal experiments demonstrated significantly increased MUC1 mRNA and protein expression in the sciatic nerves of db/db mice with diabetic neuropathy. These findings align with previous immunohistochemical studies showing accumulation of structural and extracellular matrix-related proteins around peripheral nerves in diabetic patients [33,34]. Beyond its structural role, mucin-1 has been implicated in the modulation of inflammatory and oxidative stress-related signaling pathways, including responses associated with chronic low-grade inflammation [35,36]. Such mechanisms are highly relevant to the pathogenesis of diabetic neuropathy, in which sustained neuroinflammation and oxidative injury contribute to progressive nerve damage. Taken together, these observations suggest that mucin-1, as a perineurial-derived molecule, reflects both morphological remodeling and metabolic stress in diabetic nerves, supporting its potential utility as a circulating biomarker of established diabetic peripheral neuropathy.

Clinically, mucin-1 is widely recognized as CA15-3, a biomarker routinely used in oncology, particularly for monitoring breast cancer [37]. It is important to acknowledge that CA15-3 is not a disease-specific biomarker and may be elevated in a variety of malignant and non-malignant conditions, including breast and other epithelial cancers, benign breast disease, hepatic dysfunction, and chronic inflammatory or fibrotic disorders. In the present study, individuals with known malignancies or other overt systemic diseases that could substantially influence CA15-3 levels were not included. In addition, patients with type 2 diabetes were carefully categorized, and the DPN group was restricted to individuals with diabetic peripheral neuropathy as the sole microvascular complication, thereby minimizing potential confounding effects from other diabetic complications such as nephropathy or retinopathy. Owing to its standardized assay methods, broad clinical availability, and relatively low cost, CA15-3 testing is already well integrated into routine laboratory practice [38]. In malignant conditions, serum CA15-3 concentrations typically range from 30 to several hundred U/mL, with values exceeding 100 U/mL often associated with advanced or metastatic disease [39]. In contrast, the elevation observed in our DPN cohort remained well within the non-oncologic reference range, with mean levels of approximately 17.8 U/mL compared with 9–10 U/mL in diabetic patients without neuropathy and healthy controls. This modest yet statistically significant increase suggests that mucin-1 elevation in DPN is unlikely to reflect malignant processes but rather may be associated with diabetes-related microvascular injury and perineurial structural alterations. Importantly, the potential clinical value of plasma mucin-1 may lie not only in its absolute concentration but also in longitudinal changes within the normal reference range. In the routine follow-up of patients with type 2 diabetes, a gradual rise in CA15-3 levels—even when remaining within non-oncologic limits—may signal evolving neuropathic changes before overt clinical manifestations. Accordingly, while plasma mucin-1 alone cannot establish a definitive diagnosis of DPN, it may serve as a practical screening and monitoring biomarker that complements existing tools such as the Michigan Neuropathy Screening Instrument and nerve conduction studies. Given its wide availability and established use in clinical laboratories [38], incorporating serial CA15-3 measurements into routine diabetic care could help identify patients at increased risk for neuropathy who may benefit from timely neurological evaluation and early intervention. Taken together, these findings suggest that the biomarker signal of mucin-1 observed in this study predominantly reflects stages of diabetic peripheral neuropathy that are detectable by electrophysiological assessments, supporting its potential role as a screening biomarker for clinically manifest neuropathy rather than isolated early small fiber dysfunction.

Our study has several limitations. First, patients with severe DPN were not included, limiting the ability to assess associations across the full spectrum of disease severity. Second, the assessment of DPN in this study was primarily based on electrophysiological measures, including nerve conduction velocities and sensory nerve action potentials, which predominantly reflect large fiber involvement. As a result, patients with early-stage or small fiber-predominant neuropathy, which may require specialized assessments such as intraepidermal nerve fiber density or autonomic function testing, were not specifically evaluated. Therefore, the relevance of plasma mucin-1 levels in small fiber-only neuropathy remains to be determined. Third, patients with well-controlled diabetes may have had less nerve damage compared with poorly controlled patients, potentially influencing biomarker expression. In addition, although patients with other overt microvascular complications were excluded to minimize confounding, this study did not directly compare mucin-1 levels across different diabetic microvascular complications, such as nephropathy or retinopathy, which warrants further investigation. Finally, the study was cross-sectional in design, and longitudinal studies are needed to determine whether mucin-1 predicts the onset or progression of DPN. Future studies evaluating patients with isolated small fiber neuropathy may further clarify whether mucin-1 levels exhibit intermediate changes compared with healthy individuals and patients with diabetes without neuropathy.

## 5. Conclusions

This is the first study to identify mucin-1 as a potential blood-based biomarker of diabetic neuropathy, supported by both human and animal data. The integration of proteomic screening, clinical validation, and mechanistic confirmation represents a comprehensive approach rarely achieved in biomarker discovery. Plasma mucin-1 showed significant correlations with both clinical scores and electrophysiological indices and displayed higher sensitivity for DPN than MNSI. Given its availability via CA15-3 testing, plasma mucin-1 represents a promising candidate biomarker for the early detection and monitoring of DPN. Future studies should include longitudinal analyses to determine whether mucin-1 predicts the onset or progression of DPN and whether its levels reflect the response to therapeutic interventions, such as glycemic control or neuroprotective treatments.

## Figures and Tables

**Table 1:** Characteristics of participants with T2D for screening experiment.

Characteristic	HV	T2D w/o DPN	T2D with DPN	*p*-Value
Number (male/female)	10 (5/5)	20 (10/10)	13 (5/8)	0.078
Age (year)	54.70 ± 4.32	56.70 ± 7.07	59.23 ± 7.36	0.271
Body weight (kg)	57.99 ± 8.18	61.85 ± 10.12	66.48 ± 9.64	0.116
BMI (kg/m^2^)	24.04 ± 1.93	24.48 ± 2.62	25.92 ± 2.95	0.093
Waist circumference (cm)	78.10 ± 6.99	82.50 ± 7.13	80.08 ± 6.53	0.075
Fasting glucose (mg/dL)	79.60 ± 6.38	138.35 ± 14.56	132.54 ± 15.24	<0.001
HbA1c (%)	5.29 ± 0.17	7.48 ± 1.24	7.32 ± 1.39	<0.001
Fasting insulin (µIU/mL)	3.19 ± 1.70	5.91 ± 5.04	6.94 ± 5.67	0.047
HOMA-IR	0.62 ± 0.32	2.19 ± 2.35	2.33 ± 2.11	0.038
M/C ratio	2.88 ± 1.50	9.94 ± 7.57	11.91 ± 8.75	0.012
eGFR (mL/min/1.73 m^2^)	102.57 ± 19.03	101.29 ± 19.22	95.97 ± 19.17	0.658
Creatinine (mg/mL)	0.75 ± 0.15	0.72 ± 0.15	0.73 ± 0.16	0.891
AST (U/L)	25.00 ± 6.93	27.85 ± 12.57	29.77 ± 12.31	0.616
ALT (U/L)	23.10 ± 7.92	28.60 ± 12.76	28.69 ± 12.34	0.683
Triglyceride (mg/dL)	110.40 ± 30.62	111.75 ± 22.73	123.59 ± 24.13	0.243
HDL (mg/dL)	62.02 ± 23.19	53.47 ± 12.07	50.42 ± 12.21	0.194
LDL (mg/dL)	108.15 ± 26.02	103.30 ± 28.63	83.00 ± 26.90	0.233
T2D duration (year)	0	9.60 ± 2.32	12.85 ± 5.76	0.013

Values are expressed as number or mean ± SD. BMI, body mass index; HbA1c, hemoglobin A1c; HOMA-IR, homeostatic model assessment of insulin resistance; M/C ratio, microalbumin–creatinine ratio; eGFR, estimated glomerular filtration ratio; AST, aspartate aminotransferase; ALT, alanine aminotransferase; HDL, high-density lipoprotein cholesterol; LDL, low-density lipoprotein cholesterol; HV, healthy volunteers; T2D, type 2 diabetes; DPN, diabetic peripheral neuropathy; w/o, without.

**Table 2:** Characteristics of participants with T2D for validation experiment.

Characteristic	HV	T2D w/o DPN	T2D with DPN	*p*-Value
Number (male/female)	100 (73/27)	97 (54/43)	95 (43/52)	0.142
Age (year)	59.68 ± 6.71	59.13 ± 8.59	61.11 ± 9.69	0.274
Body weight (kg)	63.05 ± 7.45	67.68 ± 13.10	66.57 ± 12.51	0.061
BMI (kg/m^2^)	24.54 ± 1.52	25.19 ± 3.54	25.58 ± 4.26	0.296
Waist circumference (cm)	79.08 ± 6.30	83.73 ± 8.80	82.73 ± 4.84	0.095
Fasting glucose (mg/dL)	88.35 ± 5.00	129.05 ± 13.54	124.29 ± 18.79	0.006
HbA1c (%)	5.28 ± 0.18	7.03 ± 0.93	7.53 ± 1.26	0.001
Fasting insulin (µIU/mL)	3.11 ± 1.66	8.43 ± 6.35	7.59 ± 4.14	0.003
HOMA-IR	0.68 ± 0.36	2.77 ± 2.47	2.72 ± 1.33	0.001
M/C ratio	2.73 ± 1.83	8.20 ± 6.52	9.15 ± 8.08	0.032
eGFR (mL/min/1.73 m^2^)	100.63 ± 19.86	102.30 ± 20.13	91.59 ± 17.69	0.553
Creatinine (mg/mL)	0.81 ± 0.18	0.79 ± 0.16	0.76 ± 0.13	0.211
AST (U/L)	21.27 ± 4.78	21.34 ± 8.19	21.80 ± 7.58	0.473
ALT (U/L)	17.09 ± 6.77	19.12 ± 9.12	19.98 ± 9.58	0.272
Triglyceride (mg/dL)	109.34 ± 32.27	119.66 ± 49.77	122.03 ± 48.48	0.062
HDL (mg/dL)	53.55 ± 12.53	50.60 ± 13.42	51.86 ± 14.11	0.061
LDL (mg/dL)	113.29 ± 29.88	118.63 ± 30.93	110.87 ± 32.50	0.093
SBP (mmHg)	118.14 ± 11.06	121.66 ± 13.65	118.02 ± 7.24	0.262
DBP (mmHg)	75.44 ± 8.46	74.36 ± 10.94	71.58 ± 7.23	0.345
T2D duration (year)	0	5.52 ± 1.66	8.27 ± 5.62	0.001

Values are expressed as number or mean ± SD. BMI, body mass index; HbA1c, hemoglobin A1c; HOMA-IR, homeostatic model assessment of insulin resistance; M/C ratio, microalbumin–creatinine ratio; eGFR, estimated glomerular filtration ratio; AST, aspartate aminotransferase; ALT, alanine aminotransferase; HDL, high-density lipoprotein cholesterol; LDL, low-density lipoprotein cholesterol; SBP, systolic blood pressure; DBP, diastolic blood pressure; HV, healthy volunteers; T2D, type 2 diabetes; DPN, diabetic peripheral neuropathy; w/o, without.

**Table 3:** Neurological characteristics and serum mucin-1 levels according to DPN status in T2D.

Characteristic	T2D w/o DPN	T2D with DPN	*p*-Value
MMSI score	0.31 ± 0.51	3.90 ± 1.14	<0.001
Mucin-1 (U/mL)	9.66 ± 2.02	17.76 ± 4.45	0.004
T2D duration (year)	5.52 ± 1.66	8.27 ± 5.62	0.001
Peroneal MNCV	51.43 ± 3.75	44.20 ± 3.96	0.001
Tibial MNCV	45.41 ± 6.70	39.56 ± 7.11	0.007
Sural SNCV	47.47 ± 3.77	38.74 ± 4.23	0.001
Sural SNAP	15.09 ± 2.69	10.00 ± 1.71	0.019

Values are expressed as number or mean ± SD. MNSI, Michigan Neuropathy Screening Inventory; MNCV, nerve conduction velocity; SNCV, sensory nerve conduction velocity; SNAP, sensory nerve action potential; T2D, type 2 diabetes; DPN, diabetic peripheral neuropathy; w/o, without.

**Table 4:** Correlations of serum mucin-1 with clinical parameters in T2D.

Variables	Mucin-1	
	Pearson Correlation Coefficient R	*p*-Value
MNSI score	0.330	0.006
Peroneal MNCV	−0.323	0.042
Tibial MNCV	−0.297	0.046
Sural SNCV	−0.351	0.039
Sural SNAP	−0.317	0.045
T2D duration (year)	0.439	<0.001
BMI (kg/m^2^)	0.049	0.687
Glucose (mg/dL)	−0.146	0.230
HbA1c (%)	0.061	0.620
M/C ratio	0.167	0.170
Insulin (μIU/mL)	−0.199	0.100
HOMA-IR	−0.186	0.126

MNSI, Michigan Neuropathy Screening Inventory; MNCV, nerve conduction velocity; SNCV, sensory nerve conduction velocity; SNAP, sensory nerve action potential; T2D, type 2 diabetes; BMI, body mass index; HbA1c, hemoglobin A1c; M/C ratio, microalbumin–creatinine ratio; HOMA-IR, homeostatic model assessment of insulin resistance; DPN, diabetic peripheral neuropathy; w/o, without.

**Table 5:** Logistic regression analysis of predictors for DPN.

Variables	B	Standard Error	Wald	Degrees of Freedom	*p*-Value	Estimated Odd Ratio	95% Confidence Interval
Mucin-1	0.569	0.248	5.271	1	0.022	1.766	1.087–2.871
MNSI score	0.861	0.424	4.122	1	0.042	2.366	1.030–5.434
Peroneal MNCV	−0.273	0.135	4.093	1	0.043	0.761	0.584–0.992
Tibial MNCV	−0.254	0.108	4.008	1	0.047	0.713	0.512–0.937
Sural SNCV	−0.266	0.127	4.051	1	0.039	0.758	0.579–0.976
Sural SNAP	−0.291	0.131	4.085	1	0.045	0.683	0.438–0.913
BMI (kg/m^2^)	−0.039	0.154	0.064	1	0.800	0.962	0.711–1.300
Glucose (mg/dL)	−0.035	0.050	0.511	1	0.475	0.965	0.876–1.064
HbA1c (%)	1.046	0.691	2.290	1	0.130	2.845	0.734–11.022
M/C ratio	−0.057	0.071	0.643	1	0.423	0.945	0.822–1.086
Insulin (μIU/mL)	−0.216	0.631	0.117	1	0.732	0.806	0.234–2.777
HOMA-IR	0.417	1.920	0.047	1	0.828	1.517	0.035–65.30
(constant)	−7.732	7.341	1.109	1	0.292	0.000	

MNSI, Michigan Neuropathy Screening Inventory; MNCV, nerve conduction velocity; SNCV, sensory nerve conduction velocity; SNAP, sensory nerve action potential; BMI, body mass index; HbA1c, hemoglobin A1c; M/C ratio, microalbumin–creatinine ratio; HOMA-IR, homeostatic model assessment of insulin resistance; T2D, type 2 diabetes.

## Data Availability

The original contributions presented in the study are included in the article/Supplementary Material. Further inquiries can be directed to the corresponding author.

## Supplementary Materials

The following supporting information can be downloaded at https://www.mdpi.com/article/10.3390/biom16010128/s1, Figure S1: Functional enrichment analysis of selected proteins; Figure S2: Relative expression levels of selected plasma proteins; Table S1: List of antibody array kit.

## Abbreviations

The following abbreviations are used in this manuscript:

DPN	Diabetic peripheral neuropathy
T2D	Type 2 diabetes mellitus
HV	Healthy volunteers
MNSI	Michigan Neuropathy Screening Inventory
MNCV	Motor nerve conduction velocity
SNCV	Sensory nerve conduction velocity
ROC	Receiver operating characteristic
AUC	Area under the curve
CA15-3	Cancer antigen 15-3
