# Supplementary material for: Plasma Mucin-1 as a Potential Biomarker for Diabetic Peripheral Neuropathy in Type 2 Diabetes
Source: Biomolecules. 2026 Jan 12;16(1):128. doi: 10.3390/biom16010128 (PMC12839277; doi:10.3390/biom16010128)
Supplement: Supplementary file 1 [file biomolecules-16-00128-s001.zip › biomolecules-4047606-supplementary.pdf]

Supplementary Materials

1. Supplementary Figures and Tables

1.1. Supplementary Figures

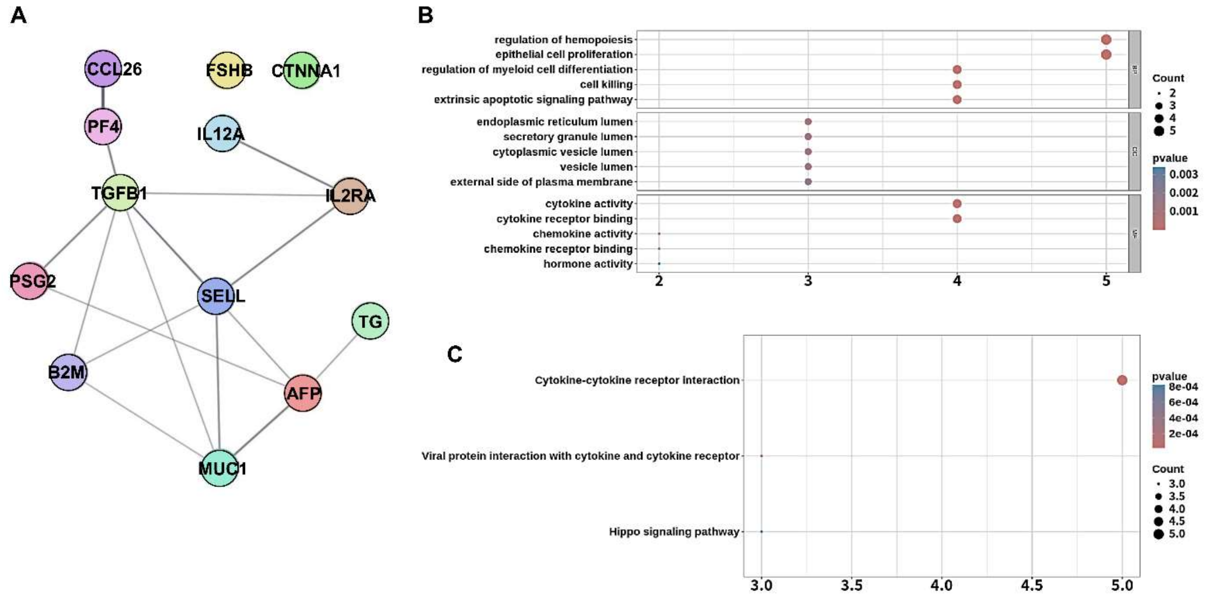

Supplementary Figure S1. Functional enrichment analysis of selected proteins. (A) PPI network of 13-significant proteins derived from antibody array analysis. The nodes indicate the proteins that connect with others through edges, showing an interaction between linked proteins. Enrichment analysis of diabetic neuropathy with GO and KEGG pathway analysis of 13-significant protein. Top five GO terms of three criteria: BP, CC, MF (B) and top three KEGG pathway (C) were illustrated respectively. The count value in x-axis is the number of significant proteins over the total proteins involved in the GO or pathway. PPI, protein-protein interaction; GO, gene ontology; MF, molecular function; CC, cellular component; BP, biological process.

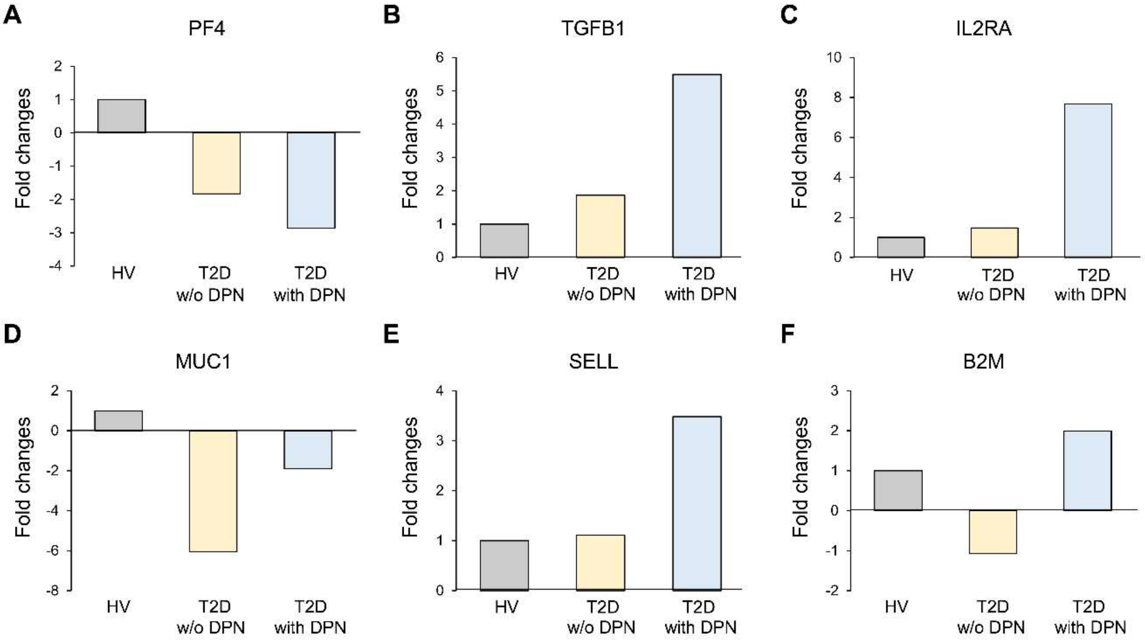

Supplementary Figure S2. Relative expression levels of selected plasma proteins. Relative expression levels of PF4 (A), TGFB1 (B), IL2RA (C), MUC1 (D), SELL (E), and B2M (F) were measured by antibody array in HV, T2D without DPN, and T2D with DPN. HV, healthy volunteers; T2D, type 2 diabetes; w/o, without; DPN, diabetic peripheral neuropathy.

1.2. Supplementary Tables

Supplementary Table S1. List of antibody array kit

ID	Antibody Name	Swiss-Prot
1	4-1BB Receptor	Q07011
2	Adiponectin	Q15848
3	EG-VEGF	P58294
4	Endostatin	P39060
5	Eotaxin	P51671
6	FGF-acidic	P05230
7	FGF-basic	P09038
8	Flt3-Ligand	P49771
9	G-CSF	P09919
10	GM-CSF	P04141

11	IGF-I	P05019
12	IGF-II	P01344
13	IL-1RA	P14778
14	IL-1α	P01583
15	IL-6	P05231
16	IL-7	P13232
17	M-CSF	P09603
18	PDGF-AA	P04085
19	PDGF-BB	P01127
20	RANTES	P13501
21	SCF	P21583
22	sFas Ligand/Apo1L	P48023
23	sIL-2Rα	P01589
24	sRANK Receptor	Q9Y6Q6
25	sRANKL	O14788
26	sTNF-receptor	P19438
27	sTNF-receptor II	P20333
28	TNF-α	P01375
29	TNF-β	P01374
30	VCAM-1	P19320
31	EGFR	P00533
32	IL-4	P05112
33	sCD40 Ligand	P29965
34	VEGF	P15692
35	IL-2	P60568
36	IL-8	P10145
37	IL-10	P22301
38	4-1BBL	P41273
39	Adipolean Variant	Q15848
40	AITRL	Q9UNG2
41	ApoE3	P02649
42	APRIL	O75888
43	Artemin	Q5T4W7

44	BAFF	Q9Y275
45	BCA-1	O43927
46	BD-1	P60022
47	BD-2	O15263
48	BD-3	P81534
49	BD-4	Q8WTQ1
50	BDNF	P23560
51	Betacellulin	P35070
52	BMP-2	P12643
53	BMP-4	P12644
54	BMP-7/OP-1	P18075
55	BRAK	O95715
56	Cardiotrophin-1	Q16619
57	CNTF	P26441
58	CTACK	Q9Y4X3
59	CTGF	P29279
60	CTGFL/WISP-2	O76076
61	CXCL16	Q9H2A7
62	EMAP-II	Q12904
63	ENA-78	P42830
64	Eotaxin-2	O00175
65	Eotaxin-3	Q9Y258
66	Exodus-2	O00585
67	FGF-10	O15520
68	FGF-16	O43320
69	FGF-17	O60258
70	FGF-4	P08620
71	FGF-5	P12034
72	Follistatin	P19883
73	Fractalkine	P78423
74	gAcrp30/Adipolean	Q15848
75	Galectin-1	P09382
76	Galectin-3	P17931

77	GCP-2	P80162
78	GDF-3	Q9NR23
79	GDNF	P39905
80	GRO/MGSA	P09341
81	GRO-β	P19875
82	GRO-γ	P19876
83	HCC-1	Q16627
84	Heregulin-β1	Q02297
85	I-309	P22362
86	IFN-β	P01574
87	IFN-λ2	Q8IZJ0
88	IGF-BP1	P08833
89	IGF-BP3	P17936
90	IGF-BP5	P24593
91	IGF-BP7	Q16270
92	IL-11	P20809
93	IL-12	P29459
94	IL-13	P35225
95	IL-15	P40933
96	IL-16	Q14005
97	IL-17 (IL-17A)	Q16552
98	IL-17B	Q9UHF5
99	IL-17D	Q8TAD2
100	IL-17E	Q9H293
101	IL-17F	Q96PD4
102	IL-19	Q9UHD0
103	IL-20	Q9NYY1
104	IL-21	Q9HBE4
105	IL-22	Q9GZX6
106	IL-3	P08700
107	IL-31	Q6EBC2
108	IL-33	O95760
109	IL-5	P05113

110	IL-9	P15248
111	IP-10	P02778
112	I-TAC	O14625
113	KGF	P21781
114	LD78β	P16619
115	LEC	O15467
116	Leptin	P41159
117	LIGHT	O43557
118	Lymphotactin	P47992
119	Maspin	P36952
120	MCP-1/MCAF	P13500
121	MCP-2	P80075
122	MCP-3	P80098
123	MCP-4	Q99616
124	MDC	O00626
125	MEC	Q9NRJ3
126	MIA	Q16674
127	MIA-2	Q96PC5
128	Midkine	P21741
129	MIG	Q07325
130	MIP-1α	P10147
131	MIP-1β	P13236
132	MIP-3	P55773
133	MIP-3α	P78556
134	MIP-3β	Q99731
135	MIP-4	P55774
136	MIP-5	Q16663
137	Nanog	Q9H9S0
138	NAP-2	P02775
139	Neuroserpin	Q99574
140	Neurturin	Q99748
141	NNT-1/BCSF-3	Q9UBD9
142	NOV	P48745

143	NP-1	P59665
144	NT-3	P20783
145	NT-4	P34130
146	Oncostatin M	P13725
147	OPG	O00300
148	PAI-1	P05121
149	Persephin	O60542
150	PF-4	P02776
151	PlGF	P49763
152	PTHrP	P12272
153	RELMβ	Q2UXL7
154	Resistin	Q9HD89
155	sCD22	P20273
156	SCGF-β	Q9Y240
157	SDF-1α	P48061
158	SDF-1β	P48061
159	sDLL-4	Q9NR61
160	sTRAIL Receptor-2	O14763
161	sTRAIL/APO2L	O50591
162	TACI	O14836
163	TARC	Q92583
164	TECK	O15444
165	TFF-2	Q03403
166	TGF-α	P01135
167	TGF-β1	P01137
168	TIMP-1	P01033
169	TL-1A	O95150
170	TPO	P40225
171	TSLP	Q969D9
172	TWEAK	O43508
173	Vaspin	Q8WXF3
174	Visfatin	P43490
175	WNT-1	P04628

176	WNT-3a	P56704
177	NGF β	P01138
178	APC	P25054
179	Catenin-α1	P35221
180	Catenin-γ	P14923
181	E-cadherin	P12830
182	Cadherin-pan	P12830
183	HER3	P21860
184	FGFR1 Oncogene Partner	O95684
185	FGFR2	P21802
186	FGFR3	P22607
187	FLI1	Q01543
188	Granzyme B	P10144
189	HDAC1	Q13547
190	HDAC10	Q969S8
191	HDAC3	O15379
192	HDAC5	Q9UQL6
193	HDAC6	Q9UBN7
194	HDAC7	Q8WUI4
195	HDAC9	Q9UKV0
196	Heregulin iso-10	Q02297
197	Integrin β5	P18084
198	MMP-1	P03956
199	MMP-10	P09238
200	MMP-11	P24347
201	MMP-13	P45452
202	MMP-14	P50281
203	MMP-15	P51511
204	MMP-16	P51512
205	MMP-19	Q99542
206	MMP-2	P08253
207	MMP-23	O75900
208	MMP-3	P08254

209	MMP-7	P09237
210	MMP-8	P22894
211	MMP-9	P14780
212	Osteopontin	P10451
213	PDGFB	P01127
214	PDGFR α	P16234
215	S100 A1	P23297
216	TGF β Receptor II	P37173
217	TGF β Receptor III	Q03167
218	TGF-β2	P61812
219	TGF-β3	P10600
220	TIMP2	P16035
221	TIMP3	P35625
222	CD40	P25942
223	FAS	P25445
224	NCoR1	O75376
225	TIMP4	Q99727
226	TRADD	Q15628
227	Tyrosinase	P14679
228	Ubiquitin	Q3MIH3
229	VEGFB	P49765
230	FER	P16591
231	Catenin-beta 1	P35222
232	STAT3	P40763
233	STAT1	P42224
234	STAT5A	P42229
235	ERCC6	Q03468
236	HER2	P04626
237	STAT5A/B	P42229
238	Apolipoprotein F (APOF)	Q13790
239	Apolipoprotein L1 (APOL1)	O14791
240	Apolipoprotein L2 (APOL2)	Q9BQE5
241	BCL-10	O95999

242	IFN-gamma	P01579
243	CD14	P08571
244	BLK	P51451
245	CIB1	Q99828
246	4E-BP1	Q13541
247	S 100A10/P11	P60903
248	C-Kit	P10721
249	PTK6	Q13882
250	LYN	P07948
251	GSK3 alpha	P49840
252	TYRO3	Q06418
253	IGF 1R	P08069
254	S 100B	P04271
255	MLL	Q03164
256	YES 1	P07947
257	GATA3	P23771
258	SRC	P12931
259	IGF-BP2	P18065
260	ALCAM	Q13740
261	AXL	P30530
262	RON	Q04912
263	SOX2	P48431
264	CD44	P16070
265	Myeloperoxidase (MPO)	P05164
266	Myostatin (GDF-8)	O14793
267	PDGFR beta	P09619
268	PROZ	P22891
269	Flt-1	P17948
270	KDR (VEGFR2)	P35968
271	ITGA5	P08648
272	EGF	P01133
273	NGFR	P08138
274	AKT2	P31751

275	GSK3 beta	P49841
276	Androgen receptor	P10275
277	EGR1	P18146
278	ICAM-1	P05362
279	GATA1	P15976
280	EPCAM	P16422
281	FAK	Q05397
282	IL-1 beta	P01584
283	AFP	P02771
284	alpha hCG	P01215
285	beta hCG	P0DN86
286	Beta-2-Microglobulin	P61769
287	CA125	Q8WXI7
288	CA15-3	Q99965
289	CA19-9	P78552
290	CEA	P11465
291	C-reactive Protein (CRP)	P02741
292	Ferritin	P02794
293	Free PSA	P07288
294	FSH	P01225
295	hCG	P01215/P01233
296	HGH	P01241
297	Insulin	P01308
298	LH (Human Luteinizing Hormone)	P01229
299	Prolactin	P01236
300	PSA-ACT	P07288
301	Thyroglobulin	P01266
302	Total PSA	P07288
303	TSH	P01222
304	Hepatocyte Growth Factor (HGF)	P14210
305	STAT6	P42226
306	E-Selectin	P16581
307	L-Selectin	P14151

308	Angiopoietin-1	Q15389
309	Angiopoietin-2	O15123
310	AKT1	P31749

H, human; M, mouse; R, rat.
